# Repetitive Exposure to Bacteriophage Cocktails against *Pseudomonas aeruginosa* or *Escherichia coli* Provokes Marginal Humoral Immunity in Naïve Mice

**DOI:** 10.3390/v15020387

**Published:** 2023-01-29

**Authors:** Chantal Weissfuss, Sandra-Maria Wienhold, Magdalena Bürkle, Baptiste Gaborieau, Judith Bushe, Ulrike Behrendt, Romina Bischoff, Imke H. E. Korf, Sarah Wienecke, Antonia Dannheim, Holger Ziehr, Christine Rohde, Achim D. Gruber, Jean-Damien Ricard, Laurent Debarbieux, Martin Witzenrath, Geraldine Nouailles

**Affiliations:** 1 Charité–Universitätsmedizin Berlin, Corporate Member of Freie Universität Berlin and Humboldt-Universität zu Berlin, Department of Infectious Diseases, Respiratory Medicine and Critical Care, 10117 Berlin, Germany; 2 Institut Pasteur, Université Paris Cité, CNRS UMR6047, Department of Microbiology, Bacteriophage Bacteria Host, 75015 Paris, France; 3 Université Paris Cité, INSERM UMR1137, IAME, 75018 Paris, France; 4 APHP, Hôpital Louis Mourier, DMU ESPRIT, Service de Médecine Intensive Réanimation, 92700 Colombes, France; 5 Department of Veterinary Pathology, Freie Universität Berlin, 14163 Berlin, Germany; 6 Department of Pharmaceutical Biotechnology, Fraunhofer Institute for Toxicology and Experimental Medicine (ITEM), 38124 Braunschweig, Germany; 7 Department of Microorganisms, Leibniz Institute DSMZ, German Collection of Microorganisms and Cell Cultures GmbH, 38124 Braunschweig, Germany; 8 German Center for Lung Research (DZL), Partner Site Charité, 10117 Berlin, Germany

**Keywords:** phage therapy, immunogenicity, adaptive and innate immunity, pneumonia

## Abstract

Phage therapy of ventilator-associated pneumonia (VAP) is of great interest due to the rising incidence of multidrug-resistant bacterial pathogens. However, natural or therapy-induced immunity against therapeutic phages remains a potential concern. In this study, we investigated the innate and adaptive immune responses to two different phage cocktails targeting either *Pseudomonas aeruginosa* or *Escherichia coli*—two VAP-associated pathogens—in naïve mice without the confounding effects of a bacterial infection. Active or UV-inactivated phage cocktails or buffers were injected intraperitoneally daily for 7 days in C57BL/6J wild-type mice. Blood cell analysis, flow cytometry analysis, assessment of phage distribution and histopathological analysis of spleens were performed at 6 h, 10 days and 21 days after treatment start. Phages reached the lungs and although the phage cocktails were slightly immunogenic, phage injections were well tolerated without obvious adverse effects. No signs of activation of innate or adaptive immune cells were observed; however, both active phage cocktails elicited a minimal humoral response with secretion of phage-specific antibodies. Our findings show that even repetitive injections lead only to a minimal innate and adaptive immune response in naïve mice and suggest that systemic phage treatment is thus potentially suitable for treating bacterial lung infections.

## 1. Introduction

Given the rising incidence of multidrug-resistant (MDR) bacterial pathogens, bacteriophages (phages) are becoming a focus of interest for treating infectious diseases [1,2]. Ventilator-associated pneumonia (VAP), one of the most common nosocomial infections in ventilated patients with a high risk of mortality, is caused mainly by MDR-bacteria [3,4]. These include *Pseudomonas aeruginosa* (*P. aeruginosa*) or *Escherichia coli* (*E. coli*), which are on the WHO list of “global priority pathogens” continuously developing antibiotic resistance [5,6].

An increasing number of in vivo studies in animal models [7,8,9], as well as human case reports [10,11,12,13,14,15], have suggested the efficacy of phage therapy over the past two decades. To date, however, only a few clinical trials of phage therapy have been attempted in humans [16,17,18,19]. These studies use virulent phages that target and infect their host bacteria with high specificity, resulting in bacterial lysis [20,21]. Compared to conventional antibiotic therapies, phages have the unique advantage of self-amplification at the site of infection and do not appear to interfere with the host microbiota [22]. However, a number of challenges are currently impeding wide-scale adoption of phage therapy in Western countries, among them the definite prove of efficacy and safety of bacteriophage therapy, and more specifically the need to select the most adequate phage(s) for each patient, the risk of phage resistance development by the target bacteria and the poor knowledge about the host’s immune response to phages [23,24].

Although the interaction of phages with the innate immune system is not fully understood yet, natural or therapy-induced immunity against therapeutic phages remains a potential concern. Since phages are, among others, an ubiquitous part of the microbiome [25,26], they may lead to the production of pre-existing antibodies. High doses or systemic application during phage therapy may further activate the host’s immune system, similar to pathogens. As the first line of defense, innate immune cells, including neutrophils, monocytes and macrophages, recognize pathogens via universal molecular patterns, such as pathogen-associated molecular patterns (PAMPs): phages can be recognized mainly through extracellular, endocytic or cytoplasmatic recognition of virus-like structures (DNA, epitopes, etc.) [27,28]. Thus, activation of the adaptive immune system with an anti-phage humoral response could lead to the production of phage-specific antibodies, which could interfere with phage treatment in a multifaceted way [29,30]. One of the most critical factors potentially limiting the efficacy of phage therapy are neutralizing antibodies, which could inhibit the ability of phages to infect bacteria by binding to the structural phage proteins involved in bacteria recognition [31]. Several studies from phage treatment in animals [32,33,34] and humans [35,36,37,38] demonstrate that anti-phage neutralizing antibody titers can increase with time, depending on the route and dose of phage administration as well as phage types, but do not necessarily diminish the effectiveness of phage therapy. Furthermore, in a murine pneumonia model of phage therapy single-phage preparations were not associated with an overstimulation of the inflammatory response [39].

In addition to a phage-specific immune response, a significant risk of phage therapy is the development of bacterial phage resistance [40]. To reduce the potential for bacteria to modify their cell surface targets, it is mainly phage cocktails instead of single phages that have come into use. Phage cocktails consisting of more than one phage have a broader range of action, as they differ in their specificity against the bacterial strain(s) and optimally target multiple distinct bacterial receptors [41,42]. Therefore, phage cocktails may prevent the outgrowth of resistant strains during treatment [43]. A broad-range cocktail was also used successfully for the treatment of biofilm-forming *P. aeruginosa*, highlighting another potential advantage of phage therapy against MDR-bacteria [8].

Although it was recently shown that the success of pulmonary phage therapy relies on the synergistic action between phages and neutrophils [44], the immune response against the phage cocktails themselves, without the confounding effects of a bacterial infection, has not yet been systematically investigated. Hence, in this study we investigated the innate and adaptive immune responses to two different phage cocktails targeting either *P. aeruginosa* or *E. coli*, two clinically highly relevant MDR-pathogens responsible for a large number of VAP cases. To avoid the confounding effects of phages infecting bacteria and pre-existing activation of the immune system by bacterial breakdown products or the bacterial infection itself, we assessed intraperitoneal phage administration in uninfected, naïve mice. Although the two phage cocktails were slightly immunogenic, even repetitive injections over 7 days led only to a minimal innate and adaptive immune response in naïve mice at either 6 h or up to 21 days after treatment start. The two phage cocktails were well-tolerated and disseminated throughout the whole organism, including the lungs. These data potentially open the way for further investigations towards the efficacy of the systemic administration of phages to treat MDR-bacteria-induced VAP.

## 2. Materials and Methods

### 2.1. Animals

All animal studies were approved by the institutional and local governmental authorities at the Charité–Universitätsmedizin Berlin and Landesamt für Gesundheit und Soziales (LaGeSo) Berlin.

Mice were housed under specific pathogen-free conditions with free access to food and water and a 12 h (h) light/dark cycle. Animal housing and experimental procedures complied with the Federation of European Laboratory Animal Science Associations (FELASA) guidelines and recommendations for the care and use of laboratory animals.

The 8–10 week old female C57BL/6J WT mice (Janvier Labs, Le Genest-Saint-Isle, France), a common model organism in preclinical research, were used. Mice were injected intraperitoneally (i. p.) with 100 µL of either active phage cocktail (anti-*P. aeruginosa* cocktail 5 × 10^7^ PFU/injection per phage, anti-*E. coli* cocktail 1 × 10^8^ PFU/injection per phage), UV-inactivated phage cocktail or buffer (saline-magnesium (SM) buffer (0.1 M NaCI, 8 mM MgSO_4_, 50 mM Tris-HCI, pH 7.2–7.5)) once or daily for 7 days (d). The mice were monitored every 24 h for body temperature, body weight and general condition according to pre-defined score sheets. At 6 h (*n =* 9 per group), 10 d (*n =* 9 per group) or 21 d (*n =* 5–6 per group), mice were euthanized with ketamine (200 mg/kg body weight) and xylazine (20 mg/kg body weight). At the 6 h and 10 d time points, intraperitoneal lavage and bronchoalveolar lavage (BAL) were performed, blood samples collected and lungs and secondary lymphoid organs (spleen; draining lymph nodes) removed for further analysis. Blood cell analysis, flow cytometry analysis and determination of plaque-forming units (PFU) were performed. At the 21 d time point, blood samples were collected and spleens were removed for histopathological analysis.

#### 2.1.1. Differential Blood Count and Plasma Preparation

Blood leukocytes and platelet counts were carried out using a Scil Vet abc Hematology Analyzer (Scil animal care company GmbH, Viernheim, Germany). In addition, whole blood was centrifuged at 15,000× *g* for 10 min at 4 °C, plasma was frozen in liquid nitrogen and stored at −80 °C until further analysis.

#### 2.1.2. Peritoneal Lavage, Bronchoalveolar Lavage and Organ Removal

Mice were subjected to deep anesthesia. Peritoneal lavage was performed by flushing the peritoneal cavity (PC) with 5 mL 1 × phosphate-buffered saline (PBS). After exsanguination via the vena cava, the tracheas were cannulated and lungs lavaged twice with 0.8 mL 1 × PBS protease inhibitor (PI) solution (cOmplete^TM^, Mini Protease Inhibitor Cocktail; Roche, Basel, Switzerland). BAL and peritoneal lavage samples were stored on ice and analyzed for PFUs.

Lavaged lungs were perfused via the right heart chamber with 10 mL 1 × PBS and homogenized in 1 mL 1 × PBS PI-solution with gentleMACS^TM^ M tubes (Miltenyi Biotec, Bergisch Gladbach, Germany). The homogenates were stored on ice for PFU analysis.

Spleens were removed and stored in 1 × PBS on ice. PFU determination was performed with the supernatant of the single-cell suspensions described below. Draining lymph nodes (inguinal, mesenteric) were collected, pooled in 1 mL PBS and stored on ice for further use.

#### 2.1.3. Isolation of Spleen and Lymph Node Leukocytes and Flow Cytometry Analysis

For isolation of leukocytes from spleen and lymph nodes, the tissue was first forced through a 70-µm cell strainer (BD, Heidelberg, Germany) using a syringe plunger. After washing the cell strainer with 1 × PBS, the resulting cell suspension was centrifuged (470× *g* for 5 min at 4 °C), the supernatant discarded and the pellet resuspended in 2 mL 1 × red blood cell lysis buffer (Santa Cruz Biotechnology, Dallas, USA) for 2 min (spleen only). Cells were washed, centrifuged, pellets resuspended in PBS/0.1% BSA buffer and analyzed by flow cytometry.

For analysis of innate immune cells, cells were blocked with anti-CD16/CD32 (BD, Heidelberg, Germany) and stained with anti-CD45 (30F11; BD, Heidelberg, Germany), anti-CD11c (HL3; BD, Heidelberg, Germany), anti-CD11b (M1/70; eBioscience, Frankfurt, Germany), anti-F4/80 (BM8; BioLegend, San Diego, USA), anti-Ly6G (1A8; BD; Heidelberg, Germany), anti-MHCII (M6/114.15.2; eBioscience; Frankfurt, Germany), anti-CD80 (16-10A1; BD; Heidelberg, Germany) and anti-CD86 (GL1; BD; Heidelberg, Germany) monoclonal antibodies (mAbs). The gating strategy for the analysis of innate immune cells is shown in Figure S1 (Supplementary Materials).

For analysis of adaptive immune cells, cells were blocked with anti-CD16/CD32 (BD; Heidelberg, Germany), surface stained with anti-CD3 (500A2; eBioscience; Frankfurt, Germany), anti-CD4 (RM4.5; BD; Heidelberg, Germany), anti-CD8α (56–6.7; eBioscience; Frankfurt, Germany), anti-CD44 (IM7; eBioscience; Frankfurt, Germany) and anti-γδTCR (GL3; BD; Heidelberg, Germany), fixed and permeabilized with FoxP3/Transcription Factor Staining Buffer Set (eBioscience; Frankfurt, Germany) and stained intranuclearly with anti-FoxP3 (FJK-16s; eBioscience; Frankfurt, Germany), anti-RORγt (B2D; eBioscience), and anti-T-bet (4B10; eBioscience; Frankfurt, Germany) mAbs. The gating strategy for the analysis of adaptive immune cells is shown in Figure S2.

All stained cells were analyzed using the BD FACS Canto II with the BD FACSDiva software. CountBright Absolute Counting Beads (Thermo Fisher Scientific, Waltham, USA) were used for calculation of total cell numbers.

#### 2.1.4. Histopathology Analysis

The spleens of mice at 21 d were carefully removed and fixed in 4% buffered formaldehyde solution for 24–48 h, embedded in paraffin and cut into 2 μm sections. After routine dewaxing and dehydration, sections were stained with GL7 (GL-7; eBioscience; Frankfurt, Germany) mAb and hematoxylin. Histopathological analysis of the germinal center B cells was performed by the Institute of Veterinary Pathology, Faculty of Veterinary Medicine, Freie Universität Berlin, Germany. The forming of germinal centers was scored into non-existent (0), minimal (1), low-grade (2), moderate (3) and intense (4). Section analysis was performed in a blinded manner.

#### 2.1.5. Anti-Phage Antibody Measurement via ELISA

Specific anti-phage antibodies (IgG, IgM, IgA) in plasma were measured by ELISA. The anti-*P. aeruginosa* phage cocktail used for these experiments were prepared from plates with confluent lysis for each individual phage. After resuspension in phage buffer, centrifugation at 1254× *g* for 15 min w/o break, phage suspensions were filtered consecutively with 0.45 µm and 0.22 µm and stored at 4 °C until further use. Flat-bottom 96-well plates (Sarstedt) were coated with the anti-*E. coli* phage cocktail (1 × 10^8^ PFU/phage) in 100 μL/well or with the anti-*P. aeruginosa* cocktail (5 × 10^7^ PFU/phage in 100 µL) overnight at 4 °C. Wells were washed 5 times with PBS with 0.05% Tween 20 (Sigma-Aldrich, St. Louis, USA) and blocked with 1% bovine serum albumin (BSA; Sigma-Aldrich, St. Louis, USA) in PBS (100 µL/well) at room temperature (RT) for 45 min. Plates were washed 5 times with PBS/0.05% Tween 20. Diluted plasma samples in duplicates were added to the wells (100 μL/well) and incubated at 37 °C for 2 h. Samples were diluted as follows: 1:10 for plasma IgM, IgA and 1:500 for plasma IgG testing for the active phage-cocktail treated mice, while 1:10 each for the UV-inactive cocktail groups and the buffer controls. After washing the plates 5 times with PBS/0.05% Tween 20, 100 µL/well of the corresponding detection antibodies were added and the plates were incubated for 1 h at RT in the dark. The following secondary antibodies were used: biotinylated goat anti-mouse IgG (ThermoFisher scientific, Waltham, USA; SA58-10239), biotinylated rabbit anti-mouse IgM (ThermoFisher scientific, Waltham, USA; SA5-10242), biotinylated rabbit anti-mouse IgA (ThermoFisher scientific, Waltham, USA; SA5-10236). The antibody solution was removed and the plates were washed 5 times with PBS/0.05% Tween 20. HRP-conjugated streptavidin solution (ThermoFisher scientific, Waltham, USA; N100) was incubated at RT in the dark for 1 h (100 µL/well). As a substrate for peroxidase, we used TMB (100 μL/well; Invitrogen, Waltham, USA). After incubating the plates for 15 min at RT in the dark, 50 µL of 2N H_2_SO_4_ (Carl Roth) was added to stop the reaction. The absorbance was measured at 450 nm using a Multiskan^TM^ FC photometer (ThermoFisher Scientific, Waltham, USA) and analyzed with SkanIt Software 4.1 for Microplate Readers RE, ver. 4.1.0.43 and normalized by subtracting the blanks (PBS instead of plasma).

#### 2.1.6. Cytokine Analysis via LEGENdplex^TM^

Soluble cytokines and chemokines in plasma samples of the 6 h, 10 d and 21 d mice were measured using the Mouse Anti-Virus Response Panel (13-plex with V-bottom plates; BioLegend, San Diego, CA, USA) and the corresponding software provided by BioLegend. Samples were analyzed once, according to the manufacturer’s recommendations.

### 2.2. Bacteriophages and Bacterial Strains

Two different bacteriophage cocktails targeting either *P. aeruginosa* or *E. coli* were used (Table 1). The phage cocktails were assembled by mixing the single phage suspensions immediately before each experiment to ensure accurate titers. The anti-*P. aeruginosa* phage cocktail includes the two *Pseudomonas* phages DSM 19872 (JG005) [45] and DSM 22045 (JG024) [46] (5 × 10^7^ PFU/phage in 100 µL) provided by the DSMZ (Braunschweig, Germany) and ITEM Fraunhofer (Braunschweig, Germany). The anti-*E. coli* phage cocktail includes the five *E. coli* phages 536_P3, CLB_P2 [47], LF110_P3, LF73_P1 and DIJ07_P1 (1 × 10^8^ PFU/phage in 100 µL) isolated, characterized and provided by Institut Pasteur (Paris, France). Single phage suspensions were highly purified and endotoxins were removed by the providing institutions. UV-inactivation was performed using a UVC 500 Ultraviolet Crosslinker (Hoefer, Inc., San Francisco, CA, USA) for 6 h at 95,000 µJ/cm^2^. Phages were stored at 4 °C. PFU analysis was performed with the *E. coli* strain AN33 (provided by Institut Pasteur, Paris, France) and the *P. aeruginosa* strain DSM 107574 (PA74) (original source provided by Prof. S. Häußler, strain code F2029; https://bactome.helmholtz-hzi.de/cgi-bin/h-phend.cgi?STAT=1&Isol=F2029, accessed on 12 December 2022 [48]). The lytic activity of each phage within the phage cocktails against the bacterial strains was evaluated in vitro and confirmed by the providing institutions.

#### 2.2.1. Plaque Assays

Blood and organ samples were serially diluted (1:10) in phage buffer shortly before performing the plaque assays, either by double agar overlay assays (anti-*E. coli* phages) or spot tests (anti-*P. aeruginosa* phages).

Bacteria cryostocks were streaked on blood agar plates (PA74) or lysogeny broth (LB) agar plates (AN33) one day before. Bacterial cultures (tryptic soy broth (TSB), PA74; LB, AN33) were prepared (20 mL) with single colonies (OD_600_ = 0.05–0.08) and cultured with shaking (220 rpm, at 37 °C) until reaching early logarithmic phase (OD_600_ = 0.2–0.3 PA74; OD_600_ = 0.1–0.2 AN33). Low melting agar (soft agar) (4 mL/glass tube) was melted in a heating block (110 °C for 10 min) and cooled down to 48 °C. For the double agar overlay assay, 100 µL of the sample dilution and 100 µL of bacterial suspension were added to the liquid soft agar (top), gently mixed and poured on agar plates (bottom). For the spot test, only 100 µL bacterial suspension was added to the liquid soft agar. Subsequently, sample dilutions were spotted (4 µL/spot) in triplicate directly on the plates. Agar plates were incubated overnight (at 37 °C, 5% CO_2_) before calculating PFU/mL. The detection limit of the overlay assay for the *E. coli* phages against AN33 was 10 PFU/mL and the detection limit of the triplicates from the spot test for the *P. aeruginosa* phages against PA74 was 83 PFU/mL. 

#### 2.2.2. Transmission Electron Microscopy Analysis of Phage Suspensions

The two phage cocktails (active and UV-inactivated; 100 µL each) were analyzed via transmission electron microscopy by the Core Facility for Electron Microscopy of the Charité Berlin. For the negative staining, carbon-coated mesh grids were hydrophilized with Alcian blue solution (1% in 1% acetic acid) followed by washing steps in dH_2_O. The grids were placed on a drop of 20 µL particle solution for 10 min. Any residue of the solution on the grids was removed with a filter paper, followed by washing in dH_2_O and finally placing the grids on a drop of freshly prepared solution of 1% aqueous uranyl acetate for 20 s. The droplets on the grids were removed with filter paper and the grids dried until use. Imaging was performed on a Zeiss Leo 906 electron microscope at 80 kV acceleration voltage equipped with a slow scan 2K CCD camera (TRS, Moorenweis, Germany).

### 2.3. Data Analysis

Data are expressed as mean ± SD. For grouped analyses, two-way analysis of variance (ANOVA) with Tukey’s multiple comparisons test was performed. Results were considered significant if P was less than 0.05. Significance levels are indicated in the figures. Statistical analysis was performed using GraphPad Prism 9 (San Diego, CA, USA). Sample sizes of individual groups are indicated in the figure legends.

## 3. Results

### 3.1. Intraperitoneally Injected Phages Reach the Lungs and Are Harmless to Naïve Mice

In this study, we evaluated the immunogenicity of two intraperitoneally (i. p.) injected phage cocktails in naïve mice. The first cocktail includes two phages targeting *P. aeruginosa* (5 × 10^7^ PFU/phage in 100 µL) and the second includes five phages targeting *E. coli* (1 × 10^8^ PFU/phage in 100 µL).

**Figure 1:**
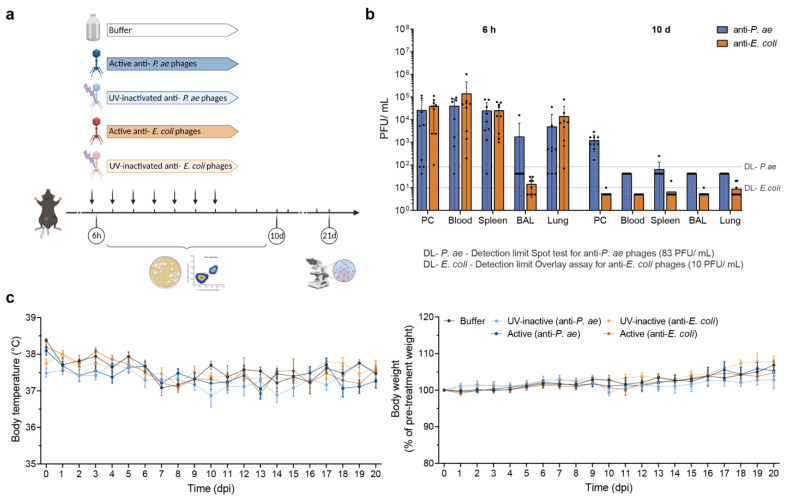
Following intraperitoneal injection, phages disseminate via blood without inducing adverse effects. (**a**) Scheme indicating experimental procedures of the study. Created with Biorender.com. Naive mice (C57BL/6J WT, female, 8–10 weeks; Janvier Labs) were treated (i. p., 100 μL) with active or UV-inactivated phage cocktails against *P. aeruginosa* or *E. coli* or buffer at 24 h intervals for 7 days. Analysis time points were 6 h, 10 d or 21 d post-injection. (**b**) Logarithmic display of phage load at 6 h and 10 d in indicated organs (peritoneal cavity (PC), blood, spleen, alveolar spaces (BAL), and lungs). DL-*P. aeruginosa*, detection limit of anti-*P. aeruginosa* phages against PA74 (83 PFU/mL); DL-*E. coli*, detection limit of anti-*E. coli* phages against AN33 (10 PFU/mL). Results are shown as mean ± SD; *n =* 9 mice per group. (**c**) Graphs displaying body temperature and body weight change over time (dpi, days post-injection). Results are shown as mean ± SEM; *n =* 9 mice per group (6 h, 10 d) or *n =* 5–6 (21 d). PMNs, polymorphonuclear neutrophils.

To determine whether the phage cocktails are recognized by the immune system of naïve mice, we first examined their distribution throughout the body at different time points. C57BL/6J wild-type mice were i. p. injected with 100 μL of either active or UV-inactivated phage-cocktail or buffer every 24 h for 7 days. Blood, spleen, lungs, bronchoalveolar lavage fluid (BAL) and i. p. lavage fluid (peritoneal cavity, (PC)) were collected at either 6 h after the first injection or 72 h after the last injection (10 d) (Figure 1a) and plaque assays were performed. Six hours following phage injection, we detected active anti-*P. aeruginosa* and anti-*E. coli* phages in all analyzed organs and compartments (PC, blood, spleen, lungs), except for the alveolar spaces (BAL). Phages in the BAL were only detected in one of nine (anti-*P. aeruginosa* phages) or five of nine (anti-*E. coli* phages) mice at this time point (Figure 1b). On day 10, we still detected anti-*P. aeruginosa* phages in the PC of all animals and one mouse carried respective phages in the spleen. Anti-*E. coli* phages were found in few individuals in the spleen (*n =* 1), BAL (*n =* 1) and lungs (*n =* 3) 10 days following treatment initiation (Figure 1b).

To check whether mice showed clinical signs of illness related to the phage treatment, we monitored them for alteration in body temperature (Figure 1c, left) and body weight (Figure 1c, right), as well as general behavior for up to 21 days after the start of treatment. Body temperature was similar in all groups and remained in a physiological range. The mice from all groups displayed an in line increase in body weight over time as expected for healthy maturing animals.

Taken together, these data indicate that i. p. injection allows for phage dissemination via the bloodstream as early as 6 h after application. Most importantly, phage treatment was well-tolerated without obvious adverse effects.

### 3.2. Innate Immune Response toward Phage Cocktails

#### 3.2.1. Blood Neutrophils and Monocytes Remain Unaffected by Phage Treatment

**Figure 2:**
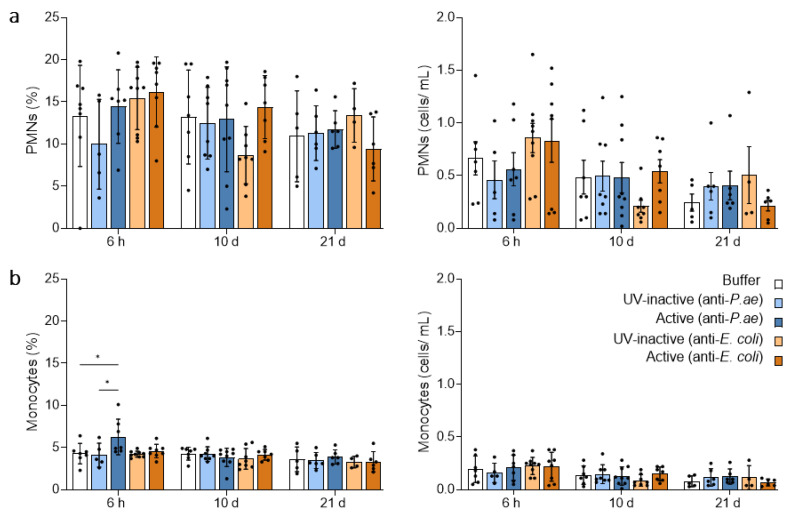
Blood PMNs remain largely unaffected by phage treatment. Bar graphs depicting the percentage and cells/mL of PMNs (**a**) and monocytes (**b**) in whole blood at 6 h, 10 d and 21 d time points as determined per complete blood count. Results are shown as mean ± SD, as determined by 2-way ANOVA with Tukey’s multiple comparisons test: * *p* < 0.05; *n =* 5–9 mice per group. PMNs, polymorphonuclear neutrophils.

To evaluate the systemic activation and sustainment of innate immunity triggered by the phage cocktails, we carried out complete blood counts at 6 h, 10 d and 21 d after start of treatment (Figure 2 and Figure S3).

Generally, the parameters, including blood leukocytes and subtypes, platelets, hematocrit (HCT) and hemoglobin (HGB), were inconspicuous and did not differ between treatment groups and controls (Figure S3). Specifically, the mobilization of polymorphonuclear neutrophils (PMNs) into blood from bone marrow, which has been shown to occur within 6 h after exposure to a substance recognized as foreign [49], was not detected upon anti-*P. aeruginosa* and anti-*E. coli* phage cocktail treatments at this time point (Figure 2a). However, we did observe a significant increase in the fraction of monocytes at 6 h after the first anti-*P. aeruginosa* phage cocktail treatment, the biological impact of this 3% increase is likely to be minor, particularly since the number of monocytes per mL blood did not change (Figure 2b). At the later time points of 10 d and 21 d after the start of treatment, no changes in innate immune cell numbers and proportions in blood were detected (Figure 2a,b).

#### 3.2.2. Lymphatic Innate Immune Cells in the Spleen and Draining Lymph Nodes Do Not Display Signs of Activation after Phage Treatment

**Figure 3:**
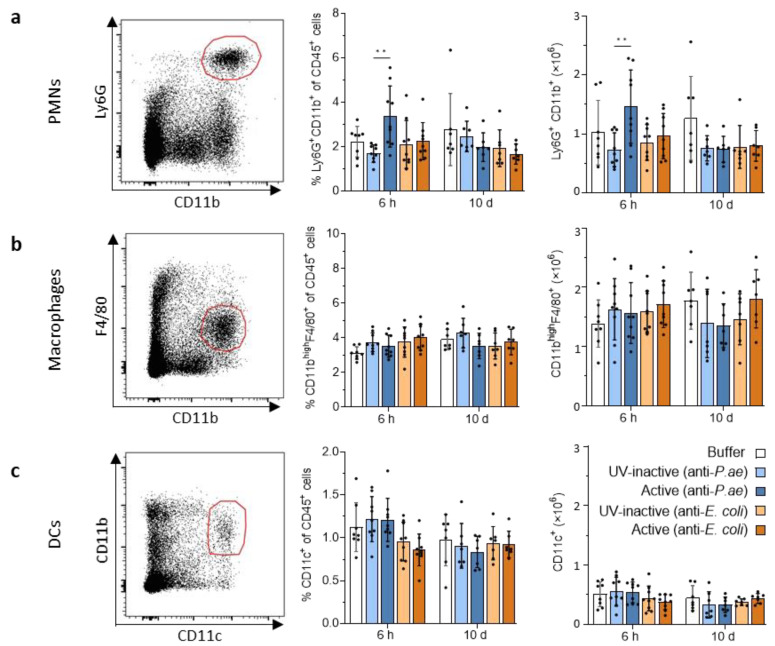
No changes in splenic innate immune cell frequencies and numbers in response to phage cocktail treatment observed. Flow cytometric analysis of (**a**) PMNs, (**b**) macrophages and (**c**) DCs in the spleen. Shown are representative dot plots (left), and bar graphs depicting the percentage of remaining cells (middle) or total cells (right). Results are shown as mean ± SD, as determined by 2-way ANOVA with Tukey’s multiple comparisons test: ** *p* < 0.01; *n =* 7–9 mice per group. For full gating strategy and cell type identifying markers see Figure S1. PMNs, polymorphonuclear neutrophils; DCs, dendritic cells.

To examine the adaptive immunity in lymphoid organs, which is initiated by activated antigen-presenting cells (APCs) loaded with antigen, we first probed spleens and draining lymph nodes for the presence of activated APCs in response to active phage cocktail encounter. Using flow cytometry for expression of costimulatory molecules and MHCII, we did not observe a significant increase in either PMNs, macrophages or dendritic cells (DCs) in the spleen at 6 h and 10 d (Figure 3a–c). PMN frequencies and total cell numbers were increased only at the 6 h time point (Figure 3a).

**Figure 4:**
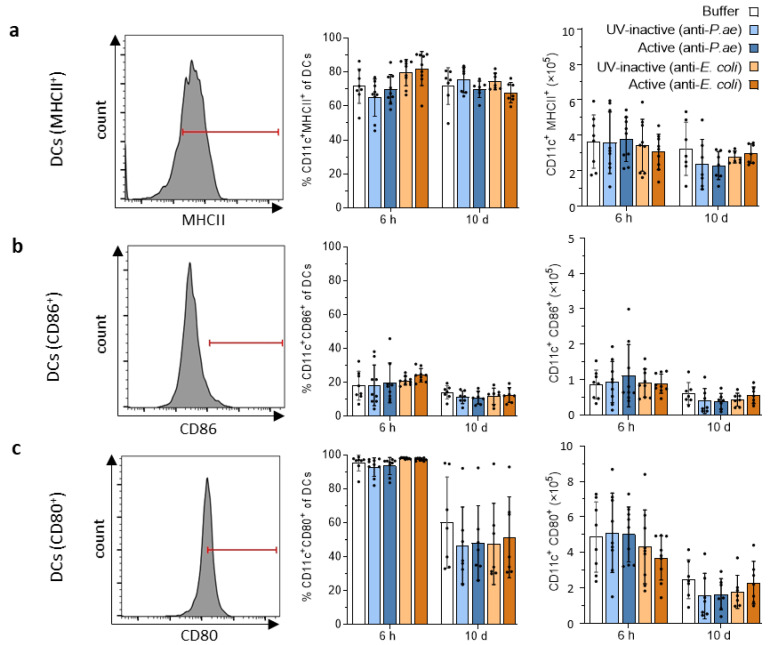
Splenic dendritic cells are not activated in response to phage cocktail treatment. Flow cytometric analysis of markers of antigen presenting cells (APC) activation (**a**) MHCII, (**b**) CD86 and (**c**) CD80 on DCs from the spleen. Shown are representative histograms (left) and bar graphs depicting the percentage of cells (middle) positive for indicated marker (MHCII, CD86, CD80) or total number of cells positive for indicated marker (right). Results are shown as mean ± SD, as determined by 2-way ANOVA with Tukey’s multiple comparisons test: *n =* 7–9 mice per group. For full gating strategy see Figure S1. Red lines define positive expression of markers based on fluorescence minus one (FMO) staining. PMNs, polymorphonuclear neutrophils; DCs, dendritic cells.

As DCs are the most competent APCs [50,51], we tested whether they upregulate MHCII on their surface as a marker of activation-induced enhanced antigen processing and presentation [52,53]. The proportion of MHCII-expressing DCs in the spleen did not increase at any of the time points analyzed, but remained in the same range as observed for the buffer and UV-inactivated phage cocktail controls (50–90%). Surface mean fluorescence intensity (MFI) of MHCII on DCs also failed to increase. MFI can be used as an equivalent to the number of MHCII molecules present on their surface, as the MFI directly correlates with the amount of stained antigen (Figure 4a). In line with this, the proportions of DCs expressing the costimulatory molecules CD80 or CD86 on their surface did not increase, nor did the MFI of CD80 or CD86 (Figure 4b,c).

Matching measurements of draining lymph nodes confirmed the absence of innate immune activation of DCs in secondary lymphoid organs at all time points analyzed (Figure S4).

### 3.3. Adaptive Immune Response against Phage Cocktails—Cellular Immunity

#### 3.3.1. Phage Treatment Does Not Trigger Adaptive T-Cell Responses in Draining Lymph Nodes or Spleen

**Figure 5:**
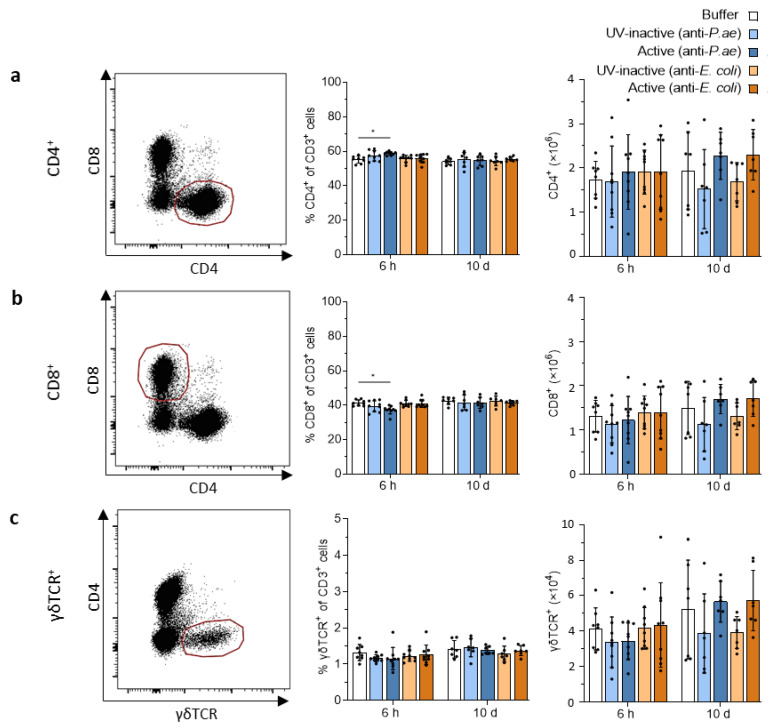
T cell populations in the draining lymph nodes show no marked changes after phage treatment. Analysis of (**a**) CD4^+^, (**b**) CD8^+^ and (**c**) γδTCR^+^ T cells in the lymph nodes. Shown are representative dot plots (left) and bar graphs depicting the percentage of remaining cells (middle) or total cells (right). Results are shown as mean ± SD, as determined by 2-way ANOVA with Tukey’s multiple comparisons test: * *p* < 0.05; *n =* 7–9 mice per group. For full gating strategy and cell type identifying markers see Figure S2.

Repetitive phage therapy carries a risk of inducing memory cells, which could be detrimental for any potential subsequent phage treatment. As a prerequisite for T-cell memory, we investigated whether repeated phage treatments induced T-cell responses in the spleen and draining lymph nodes. In lymph nodes, CD4, CD8 and γδ T cell frequencies remained similar in all groups at 6 h and 10 d and represented around 60% (a, CD4+), 40% (b, CD8+) and 1.3% (c, γδTCR+) of all T cells (CD3+) (Figure 5a–c). At 6 h post-treatment with the anti-*P. aeruginosa* phage cocktail, the proportions of CD4 T cells were moderately yet significantly increased at the expense of CD8 T cells (Figure 5a,b). We did not observe any signs of phage-induced T-cell proliferation. Repetitive treatment with phage cocktails against *P. aeruginosa* or *E. coli* did not result in an increase in CD4, CD8 or γδ T cell numbers in the draining lymph nodes at 6 h and 10 d after treatment start compared to the buffer or UV-inactivated phage cocktail controls.

Our analysis of the same T-cell populations in the spleen also failed to reveal evidence of T-cell proliferation (Figure S5). They displayed similar numbers and frequencies in all groups at both the time points analyzed.

**Figure 6:**
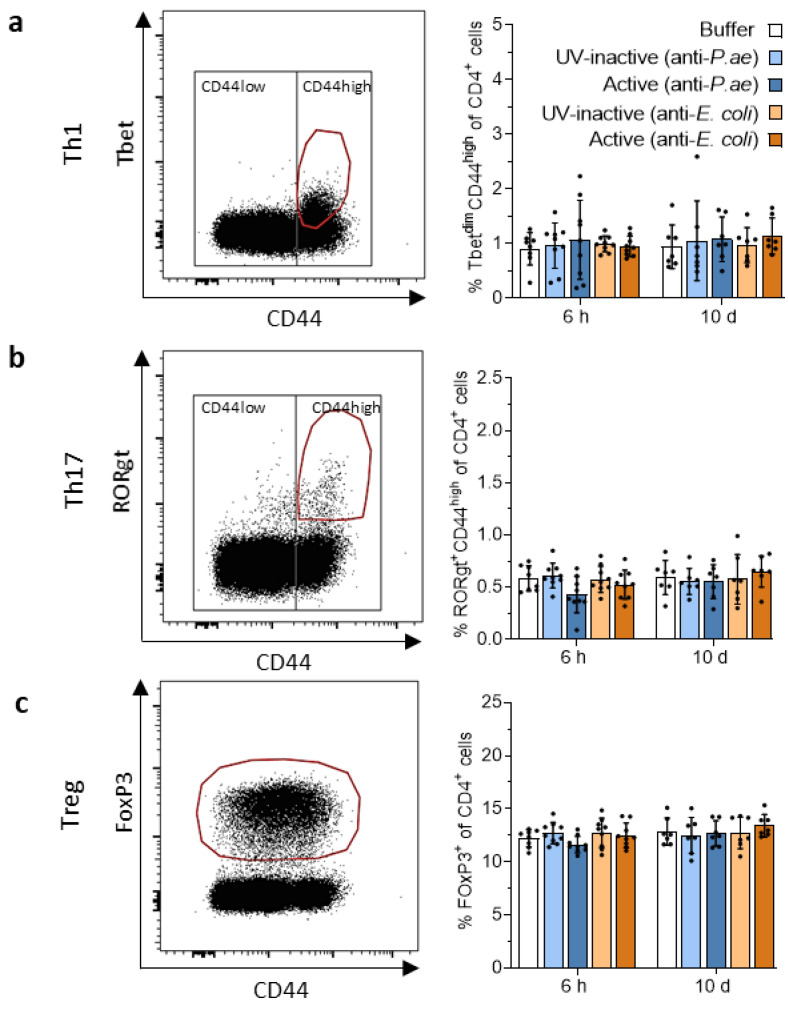
T-helper cell populations in the draining lymph nodes show no marked changes post-phage treatment. Analysis of the effector T-cell subsets (**a**) Th1, (**b**) Th17 and (**c**) Treg in the lymph nodes. Shown are representative dot plots (left) and bar graphs depicting the percentage of remaining cells (right). Results are shown as mean ± SD, as determined by 2-way ANOVA with Tukey’s multiple comparisons test: *n =* 7–9 mice per group. For full gating strategy and cell type identifying markers see Figure S2.

Despite the lack of changes in CD4, CD8 and γδ T cell numbers, we probed if CD4 effector subtypes were induced in the spleen and draining lymph nodes at 6 h and 10 d. T-helper cells type 1 (Th1) were identified by the surface expression of CD4 (CD4^+^), high expression levels of the activation marker CD44 (CD44^hi^) and intranuclear staining for the Th1-transcription factor T-bet. Only 1% of all CD4 T cells were of Th1 type and no increase in frequencies was observed in response to phage treatment at either time point (Figure 6a). Th17 cells were identified by CD4^+^, CD44^hi^ and intranuclear expression of the Th17-transcription factor RORγt. The proportion of Th17 cells among CD4 T cells was around 0.5%. As for Th1 cells, we did not observe changes in response to phage treatment at either time point (Figure 6b). Regulatory T cells (Tregs) are characterized by CD4 and transcription factor FoxP3 expression. We did not discriminate between natural and peripheral Tregs [54]. Their frequencies were around 12% of CD4 T cells and remained unaffected by phage cocktail treatments at 6 h and 10 d (Figure 6c). As observed for CD4, CD8 and γδ T cells, the findings were similar in the spleen (Figure S5).

#### 3.3.2. Minimal Pro-Inflammatory Cytokine and Chemokine Secretion after Phage Treatment

The activation of innate and adaptive immune cells and the strength of initiated immune responses rely on cytokine and chemokine milieus [55,56]. Using the Mouse Anti-Virus Response Panel (13-plex, Legendplex^TM^; BioLegend, San Diego, CA, USA), soluble cytokines and chemokines were analyzed in plasma samples at 6 h, 10 d and 21 d after the first injection (Figure S6). Six hours after treatment with the active anti-*P. aeruginosa* phage cocktail, the pro-inflammatory factors CXCL10, CCL2, CCL5 and IL-1β, were significantly increased, while treatment with the active anti-*E. coli* phage cocktail led to a significant increase in IL-6 and IL-10. At 10 d, IFN-γ levels were increased in the UV-inactivated anti-*P. aeruginosa* group compared to the buffer control or the active phage cocktail group. However, these changes may be reflective of individual outliers and were generally low.

### 3.4. Adaptive Immune Response against Phage Cocktails—Humoral Immunity

#### 3.4.1. Phage Treatment Induces Minimal to Low-Grade Germinal Center Formation

**Figure 7:**
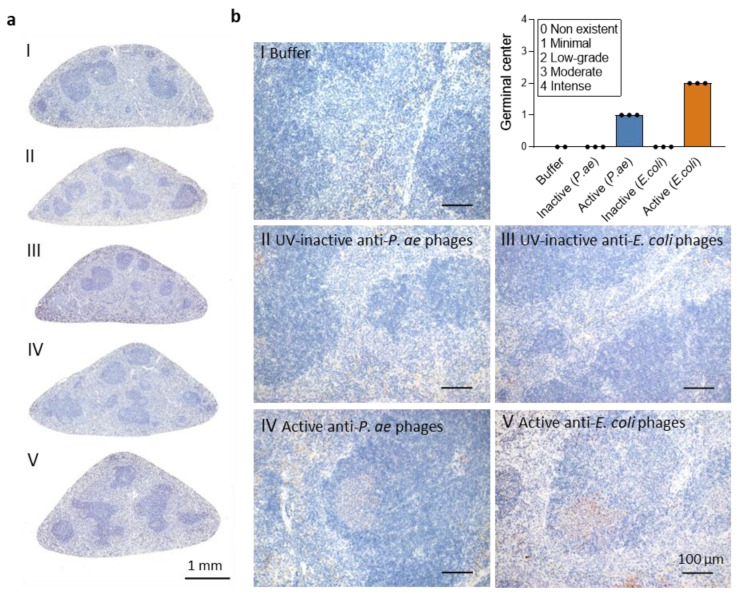
Phage treatment induces minimal to low-grade germinal center formation. Immunohistochemical staining of germinal center B cells (αGL-7 antibody; red) and hematoxylin (blue). Representative spleen sections (**a**) and corresponding enlarged images ((**b**), 20×) from animals at 21 d after start of treatment with (I) buffer, (II) UV-inactive anti-*P. aeruginosa* phage cocktail, (III) UV-inactive anti-*E. coli* phage cocktail, (IV) active anti-*P. aeruginosa* phage cocktail and (V) active anti-*E. coli* phage cocktail. Bar graph showing germinal center formation scores; *n =* 2–3 mice per group.

Humoral immunity, particularly neutralizing antibodies, could impair phage therapy. We therefore investigated if signs of germinal center reaction were visible at 21 d after treatment start, which would indicate the initiation of humoral B cell responses. Immunohistochemistry identified differences between the individual treatment groups. Spleens from mice that received buffer (I), UV-inactivated anti-*P. aeruginosa* phage cocktail (II) or UV-inactivated anti-*E. coli* phage cocktail (III) did not show any specific positive signals. In contrast, both the active phage cocktail groups (IV, V) showed minimal to minor positive signals for germinal center B cells stained by GL7 (Figure 7). These two groups also showed morphological evidence of an incipient formation of follicular centers.

#### 3.4.2. Repetitive Phage Treatment Evokes Phage-Specific Antibody Response

**Figure 8:**
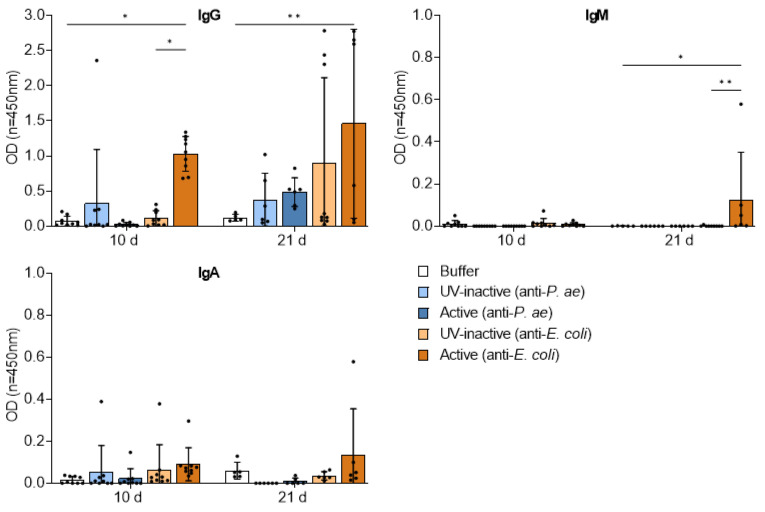
Repetitive phage treatment leads to anti-phage antibodies in plasma. Binding of plasma IgG, IgM and IgA to the active phage cocktails on 10 d and 21 d, as measured by ELISA. Results are shown as mean ± SD, as determined by 2-way ANOVA with Tukey’s multiple comparisons test: * *p* < 0.05. ** *p* < 0.01; *n =* 5–9 mice per group.

The observed formation of minimal splenic germinal centers prompted us to further investigate the humoral immune response against the two phage cocktails. To this end, we measured phage-specific antibodies (IgG, IgM, IgA) in plasma by ELISA (Figure 8).

In congruence with the presence of germinal centers, the active anti-*E. coli* phage cocktail induced an increase in IgG, IgM as well as IgA by 21 days compared to the buffer or UV-inactivated control, which for IgG was already evident by 10 d. In contrast, the treatment with the active anti-*P. aeruginosa* phage cocktail only induced a marked increase in IgG and only at the 21 d time point, but this did not reach significance. A small increase in IgG was also induced by the UV-inactivated phage cocktails but may be reflective of individual outliers. These findings suggest that the repetitive injection of each of the two phage cocktails initiated a minimal humoral B-cell response with secretion of phage-specific antibodies.

#### 3.4.3. Destruction of Phage Structures by UV-Inactivation

For evaluating the immunogenicity of the two phage cocktails, we assumed that the immune response against the UV-inactivated and the active phages would be comparable, as we expected the UV treatment to merely cross-link phage DNA [57]. To understand the low to absent humoral response against the UV-inactivated phage cocktails (Figure 7 and Figure 8), we performed negative-staining transmission electron microscopy of all phage preparations (Figure S7). In the active phage cocktails, virus-like structures were identified (Figure S7a,b). In contrast, following UV-inactivation, only artifacts that may be fragments of phages could be found (Figure S7c,d).

## 4. Discussion

Phage treatment of bacterial lung infections may be developing into an effective resource in the fight against the increasing antibiotic resistance of clinically relevant pathogens. As natural or therapy-induced immunity against therapeutic phages remains a safety and efficacy concern, in this study we assessed the immunogenicity of two phage cocktails targeting either *P. aeruginosa* or *E. coli* in naïve mice without the confounding effects of a bacterial infection. Our results show that phages reached the lungs after systemic injection. Even repetitive exposure to the phage cocktails led to only a minimal innate and adaptive immune response, while all mice remained healthy without evidence of any adverse effects.

Assessing the possible immune responses towards the two different phage cocktails, we did not observe a significant activation of innate immune cells in blood or lymphoid organs at 6 h after treatment, except for splenic PMNs in response to the active anti-*P. aeruginosa* phage cocktail. There was no upregulation of MHCII or co-stimulatory molecules on DCs that would indicate maturation into APCs and only a minimal pro-inflammatory cytokine profile at this time point. Despite the lack of evidence for an early innate response, we did observe a minimal adaptive immune response. Although we failed to observe the activation of main T cells or sub-populations at 10 days post-treatment, the histopathology analysis at 21 days nonetheless revealed a minimal to low-grade germinal center formation with increasing levels of phage-specific antibodies in plasma. The active anti-*E. coli* cocktail induced IgG and lower levels of IgA already at 10 days and IgM at 21 days, while the anti-*P. aeruginosa* phages induced only IgG and only at the late point of 21 days.

Phages can induce pro- and anti-inflammatory responses, depending on phage type, the route and duration of administration and the overall amount of phage virions present in the organism [58,59]. Consistent with our results, minimal cytokine release induced by phage treatment has also been reported in several other studies, indicating promising signs of immunogenic tolerance. Low cytokine levels after prophylactic phage treatment by intraperitoneal application were also reported in a murine *A. baumannii* infection study [60] or after the oral application of bacteriophage T7 for 10 days [61]. Differences due to the application routes of phages were demonstrated in a murine efficacy study of two *A. baumannii* phages, where intraperitoneal application resulted in the most pronounced, yet still low, immune response compared to oral or intranasal application [62].

Our results of increasing IgG titers and decreasing IgM titers over time are consistent with an antibody class switch. Similar findings, as well as weaker systemic IgA titers at later time points, have been shown by other studies [29,33]. IgA is the predominant antibody in mucosal immunity [63,64]. It is induced upon oral or intranasal immunization with bacteria [65,66] or even oral application of phages: Majewska et al. reported an IgA rise after the oral application of T4 phages in a long-term study [37] and after therapeutic administration of two staphylococcal phages mixed with the drinking water [33]. Interestingly, secreted phage-specific IgA appeared to be the major factor limiting phage activity in the gut, but the authors also noted that once the phage was removed from the diet, secretory IgA decreased over time [33]. We did not measure phage-specific IgE, which would indicate an allergic reaction. However, Cha et al. could show only a low increase in IgE production in serum upon daily intraperitoneal injection of an anti-*A. baumannii* phage cocktail [62].

Although we did find some evidence of a weak antibody response to our phage cocktails, antibodies do not necessarily have neutralizing capacity—the main concern regarding the immune response to therapeutic phages—since phage-specific neutralizing antibodies could diminish therapeutic success. However, although the presence of phage-specific antibodies with neutralizing capacity, in particular IgG2a and IgG2b, was detected after repeated intraperitoneal applications of an *A. baumannii*-specific phage cocktail in naïve mice, this did in fact not negatively affect the efficiency of the phages in a wound infection model [60]. Antibody formation and neutralizing capacity depend on the phage type, especially the capsid and tail protein composition; therefore, phages vary in immunogenicity and can induce different antibody responses [58,59,67]. In a murine study, Chechushkov et al. [67] found that phages with podovirus morphotype were non-immunogenic. In contrast, they observed an increase in neutralizing antibodies after triple immunization with phages of myovirus and siphovirus morphotypes [67]. Additionally, Majewska et al. identified multicopy proteins of phages [33], the T4 phage proteins Hoc, Soc and gp12 [37,68], as well as the conserved structural proteins gp22 and gp29 of *Pseudomonas* phages [69] as highly immunogenic. In our study, the two *P. aeruginosa* phages belonged to the myovirus morphology, as well as four *E. coli* phages, the fifth belonging to the podovirus morphotype. In addition, our findings, as well as those of other groups [33,37] align with the significant rise in neutralizing antibodies observed after 3 weeks in a rabbit model [32]. Thus, there appears to be a “therapeutic window” of phage treatment prior to the production of phage-specific antibodies [29]. Whether the antibodies observed in our model have a neutralizing capacity has to be investigated further, ideally in a murine VAP model, which will also allow analysis of any effects on therapeutic efficacy. So far, however, most animal [32,33,34] and human [36,37,38,70] studies that have investigated antibody responses to phages found that they do not interfere with phage therapy.

Natural antibodies result from continuous contact with endogenous phages, which make up a significant part of the mammalian virome [26,71]. If such pre-existing natural antibodies against phages in our cocktails were present in the mice, we would have expected to observe them even in the groups that received the UV-inactivated phage cocktails or buffer only. Hodyra-Stefaniak et al. reported natural antibodies against PB1-related *Pseudomonas* phages [69] as well as *Staphylococcus* phages [15] in the serum of healthy human volunteers. However, they had only a low neutralizing capacity. A rapid increase in IgG levels after initial immunization may indicate the previous contact of experimental mice with the applied phages [67]. Conversely, naturally present phages in the gut may lead to immunological tolerance [59]. Priming the immune system with phages from the microbiome could explain the stronger reaction to the *E. coli* phage cocktail we observed in the present study.

However, the different responses to the different phage cocktails may not root in the immunogenicity of the phages themselves but rather in technical aspects: the *E. coli* cocktail consisted of five different phages each at 1 × 10^8^ PFU/phage in 100 µL, while the *P. aeruginosa* phage cocktail contained only two each at 5 × 10^7^ PFU/phage in 100 µL, resulting in a higher total phage sum applied for the *E. coli* cocktail. Endotoxins and other bacterial components that remain in the solution during phage production may elicit immune responses by themselves [72]. Notably, both phage cocktails were highly purified, however the finally applied mix of active *E. coli* phages had a lower endotoxin level compared to the anti-*P. aeruginosa* phage cocktail, indicating that in our study endotoxin concentration did not correlate with immunogenicity. Despite the two different protocols, the immune responses to each phage cocktail remain globally weak showing that different processes can lead to similar outcomes. The absence of humoral immunity observed with UV-inactivated phages is elucidated by the EM analysis. It revealed that the UV-inactivation protocol led to major damage to viral particles. Thus, intact phages seem to elicit minimal humoral-immune responses, while phage fragments alone seem non-immunogenic.

Our study has some limitations. Since we chose three different time points to study the possible early innate and late adaptive immune responses, we might have missed some responses that occurred in between. We also did not extend our analysis beyond 3 weeks, which may have provided insight into the later humoral response. In addition, we did not investigate B- and T-cell memory, which are the main parameters of rapid induction of phage-specific antibodies following phage treatment after a second interval. Moreover, our study is limited to the two phage cocktails investigated here with their corresponding phage concentration. Preparations with lower or higher phage content might induce differential immune responses. In our study, we used naïve mice under SPF conditions without a bacterial infection, to avoid any confounding immune effects by the infection itself. However, the presence of bacteria may indirectly affect the immunogenicity of phages as well. Phage-induced bacterial lysis can further boost inflammation via bacterial particles and PAMPs, as well as cell-free phage DNA and higher numbers of phage virions released locally [58], potentially acting adjuvant-like and triggering the immune system to perceive phages as foreign [73]. However, during phage treatment in a murine pneumonia model caused by pathogenic *E. coli* strains, the rapid lysis of bacteria by phages did not increase the innate inflammatory response compared to that observed after antibiotic treatment [39]. Nevertheless, the interaction of phages with immune cells during an active infection has to be further investigated since a possible synergistic mechanism of phages with innate immune cells has been reported [44].

## 5. Conclusions

In the context of lung infections with MDR-bacteria, phage therapy is of great interest for treating VAP patients. While recent studies used intratracheal applications [74] or nebulized phage suspensions [7,75,76], here we show that systemic applications of two different phage cocktails are suitable for the dissemination of phages to the lung. The phage cocktails are well-tolerated and do not induce a marked immune response in naïve mice warranting their future evaluation as antibacterial therapeutics.

## Figures and Tables

**Table 1:** Phages used in this study.

Bacteriophage	Morphology	Phage Titer in Cocktail (PFU/Injection *)	Endotoxin Level (EU/Injection *)	Indicator Strain	Analysis Strain	Reference
DSM 19872 (JG005)	*Myovirus*	5 × 10^7^	4.30 × 10^1^	F2230	DSM 107574 (PA74)	[45]
DSM 22045 (JG024)	*Myovirus*	5 × 10^7^	8.08 × 10^−1^	DSM 19882 (PA14)	DSM 107574 (PA74)	[46]
536_P3	*Podovirus*	1 × 10^8^	2.93 × 10^−2^	536	AN33	-
CLB_P2	*Myovirus*	1 × 10^8^	9.25 × 10^−1^	55989	AN33	[47]
LF110_P3	*Myovirus*	1 × 10^8^	1.58 × 10^−1^	LF110	AN33	-
LF73_P1	*Myovirus*	1 × 10^8^	3.73 × 10^−3^	LF73	AN33	-
DIJ07_P1	*Myovirus*	1 × 10^8^	1.49 × 10^−3^	DIJ07	AN33	-

* 100 µL phage cocktail was injected.

## Data Availability

Not applicable.

## Supplementary Materials

The following supporting information can be downloaded at: https://www.mdpi.com/article/10.3390/v15020387/s1. Figure S1. Exemplary flow cytometric-gating strategy for the analysis of innate immune cells in secondary lymphoid organs; Figure S2. Exemplary flow cytometric gating strategy for analysis of adaptive immune cells (T cells) in secondary lymphoid organs; Figure S3. Blood cells remain unaffected by phage treatment; Figure S4. No changes of innate immune cell frequencies and numbers in draining lymph nodes in response to phage cocktail treatment observed; Figure S5. T-cell populations in the spleen show no marked changes after phage treatment; Figure S6. Minimal cytokine release in response to phage treatment; Figure S7. Negative staining transmission electron microscopic images of phage cocktails reveal that UV-inactivation may lead to destroyed phage structures.
