# Supplementary material for: Repetitive Exposure to Bacteriophage Cocktails against Pseudomonas aeruginosa or Escherichia coli Provokes Marginal Humoral Immunity in Naïve Mice
Source: Viruses. 2023 Jan 29;15(2):387. doi: 10.3390/v15020387 (PMC9964535; doi:10.3390/v15020387)
Supplement: Supplementary file 1 [file viruses-15-00387-s001.zip › Supplementary Material_Weissfuss_Viruses-2139752-proofed_clear version.pdf]

Supplementary Materials

Repetitive Exposure to Bacteriophage Cocktails against *Pseudomonas aeruginosa* or *Escherichia coli* Provokes Marginal Humoral Immunity in Naïve Mice

Chantal Weissfuss[1*], Sandra-Maria Wienhold[1], Magdalena Bürkle[1], Baptiste Gaborieau[2,3,4], Judith Bushe[5], Ulrike Behrendt[1], Romina Bischoff[1], Imke H. E. Korf[6], Sarah Wienecke[6], Antonia Dannheim[6], Holger Ziehr[6], Christine Rohde[7], Achim D. Gruber[5], Jean-Damien Ricard[3,4], Laurent Debarbieux[2], Martin Witzenrath[1,8], and Geraldine Nouailles[1*]

*Correspondence: chantal.weissfuss@charite.de (C.W.), geraldine.nouailles@charite.de (G.N.)

Supplementary Materials

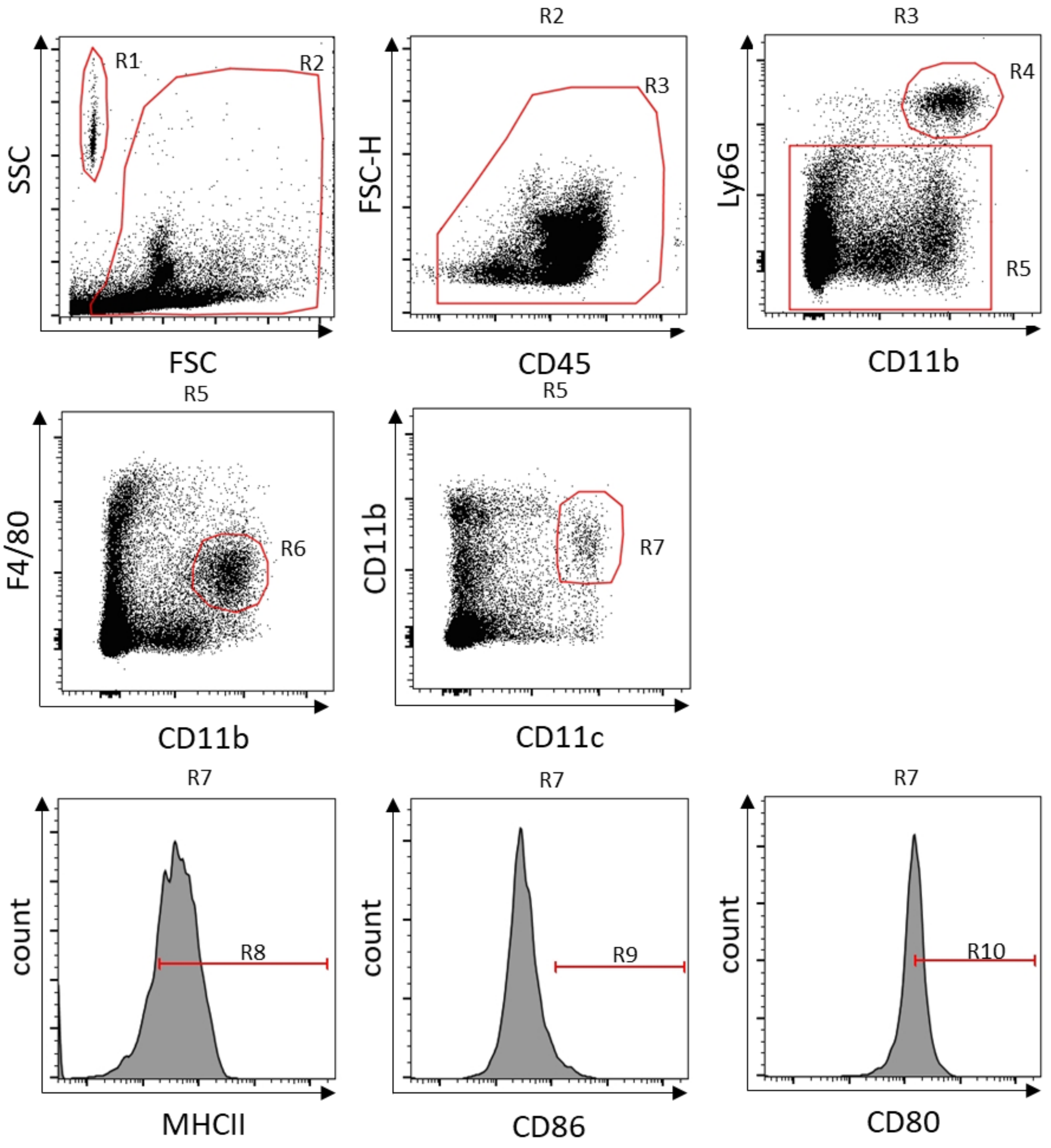

R1 Beads
R2 Cells minus debris
R3 CD45+ cells (**Leucocytes**)
R4 $CD45^+$ $Ly6G^+$ $CD11b^+$ cells (**PMNs**)
R5 $CD45^+$ $Ly6G^-$ cells
R6 $CD45^+$ $Ly6G^-$ $F4/80^+$ $CD11b^{high}$ cells (**Macrophages**)
R7 $CD45^+$ $Ly6G^-$ $CD11b^+$ $CD11c^+$ cells (**DCs**)
R8 $CD45^+$ $Ly6G^-$ $CD11b^+$ $CD11c^+$ $MHCII^+$ cells (**matDCs**)
R9 $CD45^+$ $Ly6G^-$ $CD11b^+$ $CD11c^+$ $CD86^+$ cells
R10 $CD45^+$ $Ly6G^-$ $CD11b^+$ $CD11c^+$ $CD80^+$ cells

Figure S1. Exemplary flow cytometric gating strategy for the analysis of innate immune cells in secondary lymphoid organs. Representative dot plots illustrating the gating strategy of innate immune cells in spleens and draining lymph nodes. Red lines define positive expression of markers based on fluorescence minus one (FMO) staining. PMNs, polymorphonuclear neutrophils; DCs, dendritic cells; matDCs, mature DCs.

Supplementary Materials

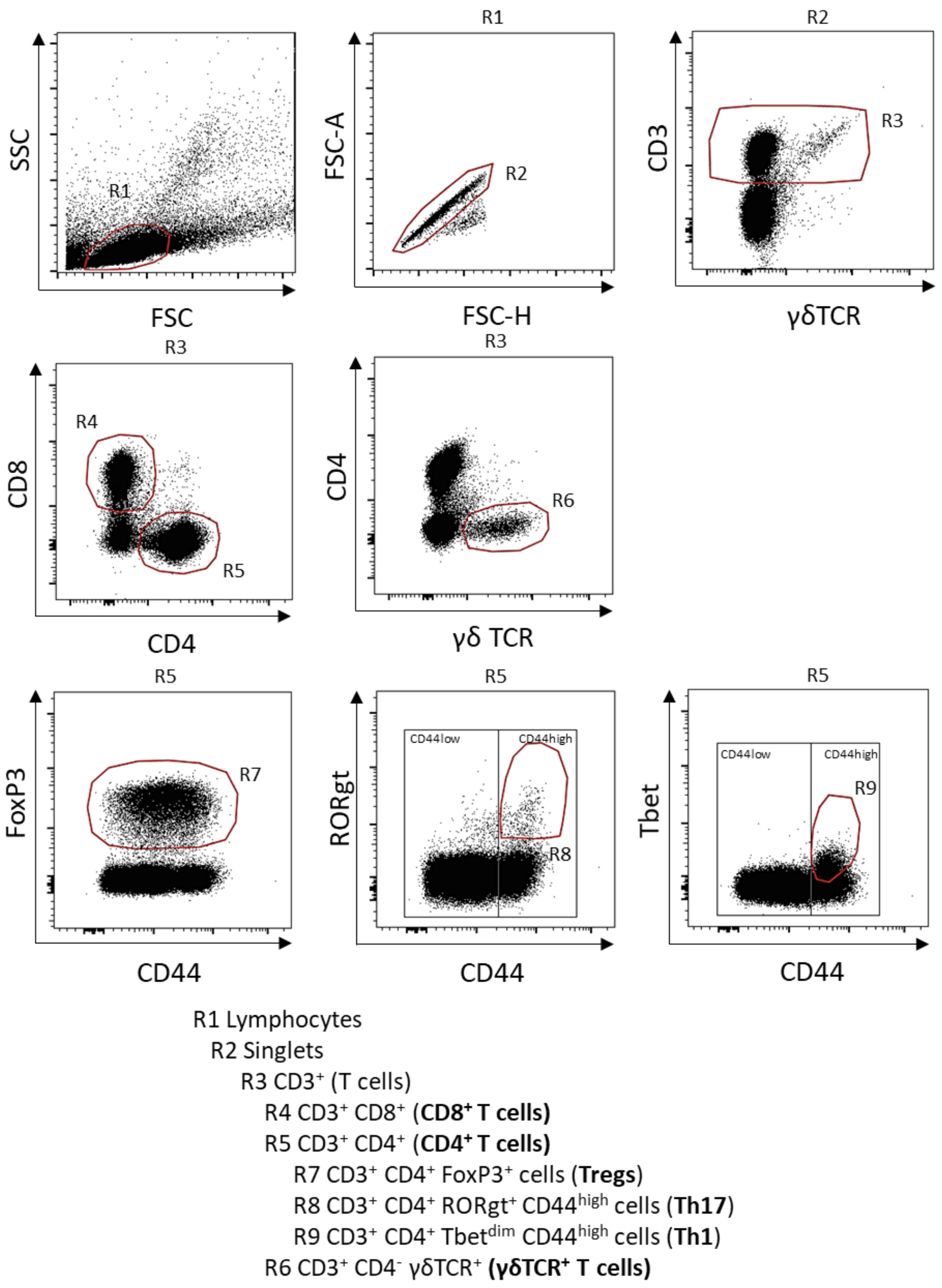

Figure S2. Exemplary flow cytometric gating strategy for analysis of adaptive immune cells (T cells) in secondary lymphoid organs. Representative dot plots illustrating the gating strategy of T cell populations in spleens and draining lymph nodes. Treg, regulatory T cells; Th17, T helper cells type 17; Th1, T helper cells type 1.

Supplementary Materials

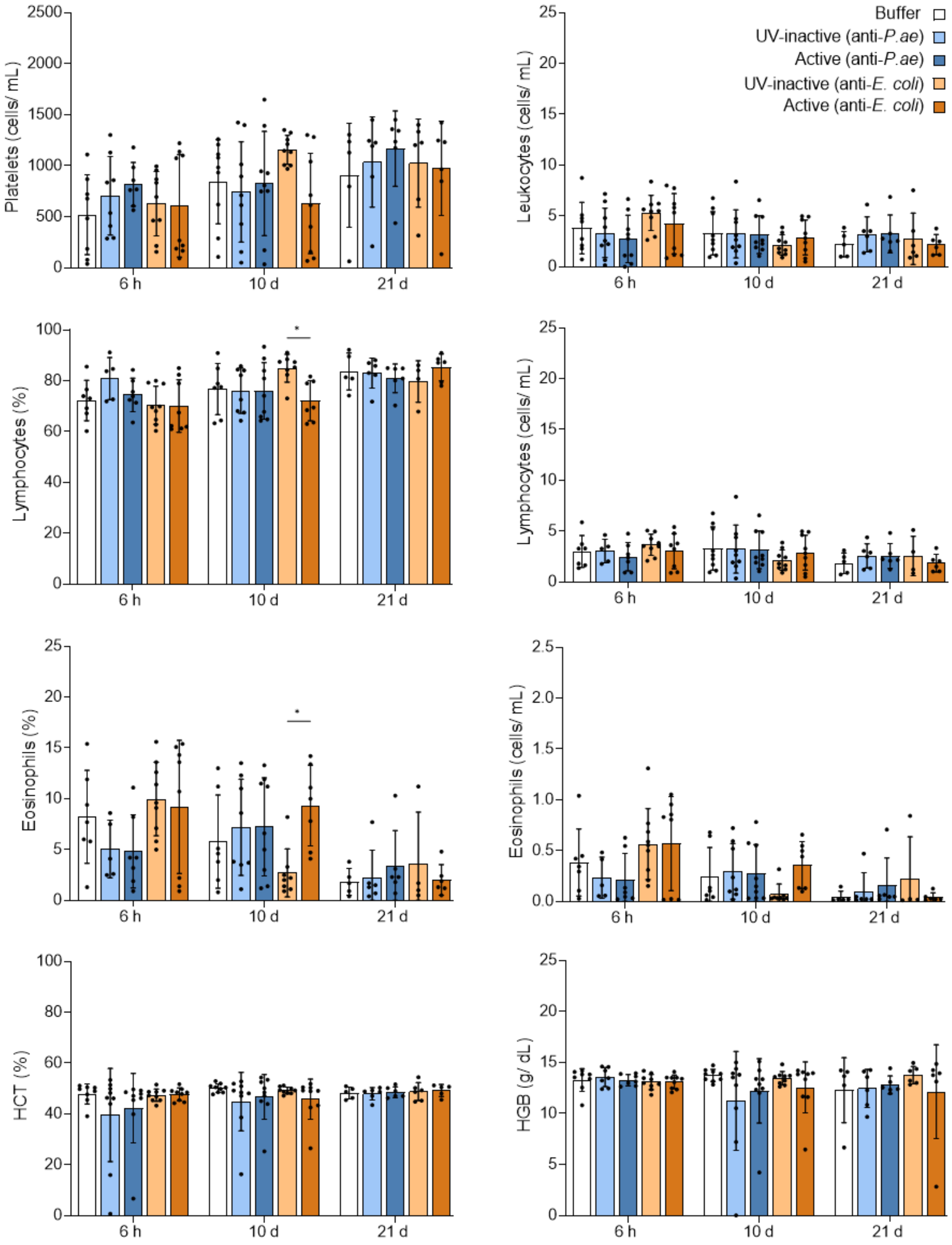

Figure S3. Blood cells remain unaffected by phage treatment. Bar graphs depicting the percentage and cells/ mL of leukocytes, platelets, lymphocytes and eosinophils as well as the hematocrit (HCT) and hemoglobin (HGB) in whole blood at 6 h, 10 d and 21 d time points as determined per complete blood count. Results are shown as mean ± SD, as determined by 2-way ANOVA with Tukey´s multiple comparisons test: *$p < 0.05$. n = 5 – 9 mice per group.

Supplementary Materials

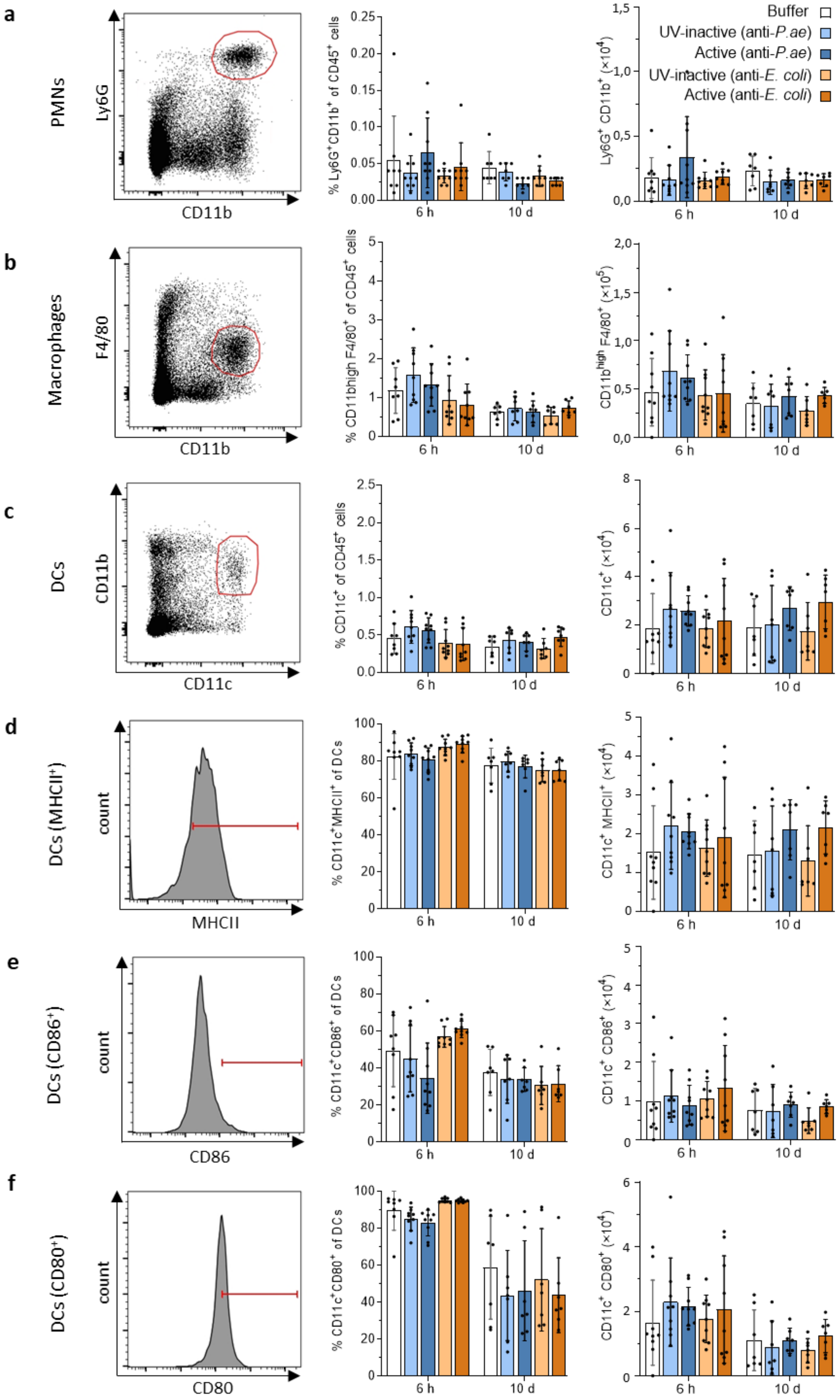

Supplementary Materials

Figure S4. No changes of innate immune cell frequencies and numbers in draining lymph nodes in response to phage cocktail treatment observed. Flow cytometric analysis of (a) PMNs, (b) macrophages, (c) DCs and analysis of markers of antigen presenting cells (APC) activation (d) MHCII, (e) CD86 and (f) CD80 on DCs in draining lymph nodes. Shown are representative dot plots (left; a-c), histograms (left; d-f) and bar graphs depicting the percentage of remaining cells (middle) positive for indicated marker (MHCII, CD86, CD80) or total cells positive for indicated marker (right). Results are shown as mean ± SD, as determined by 2-way ANOVA with Tukey´s multiple comparisons test: n = 7 – 9 mice per group. For full gating strategy see Figure S1. Red lines define positive expression of markers based on fluorescence minus one (FMO) staining. PMNs, polymorphonuclear neutrophils; DCs, dendritic cells.

Supplementary Materials

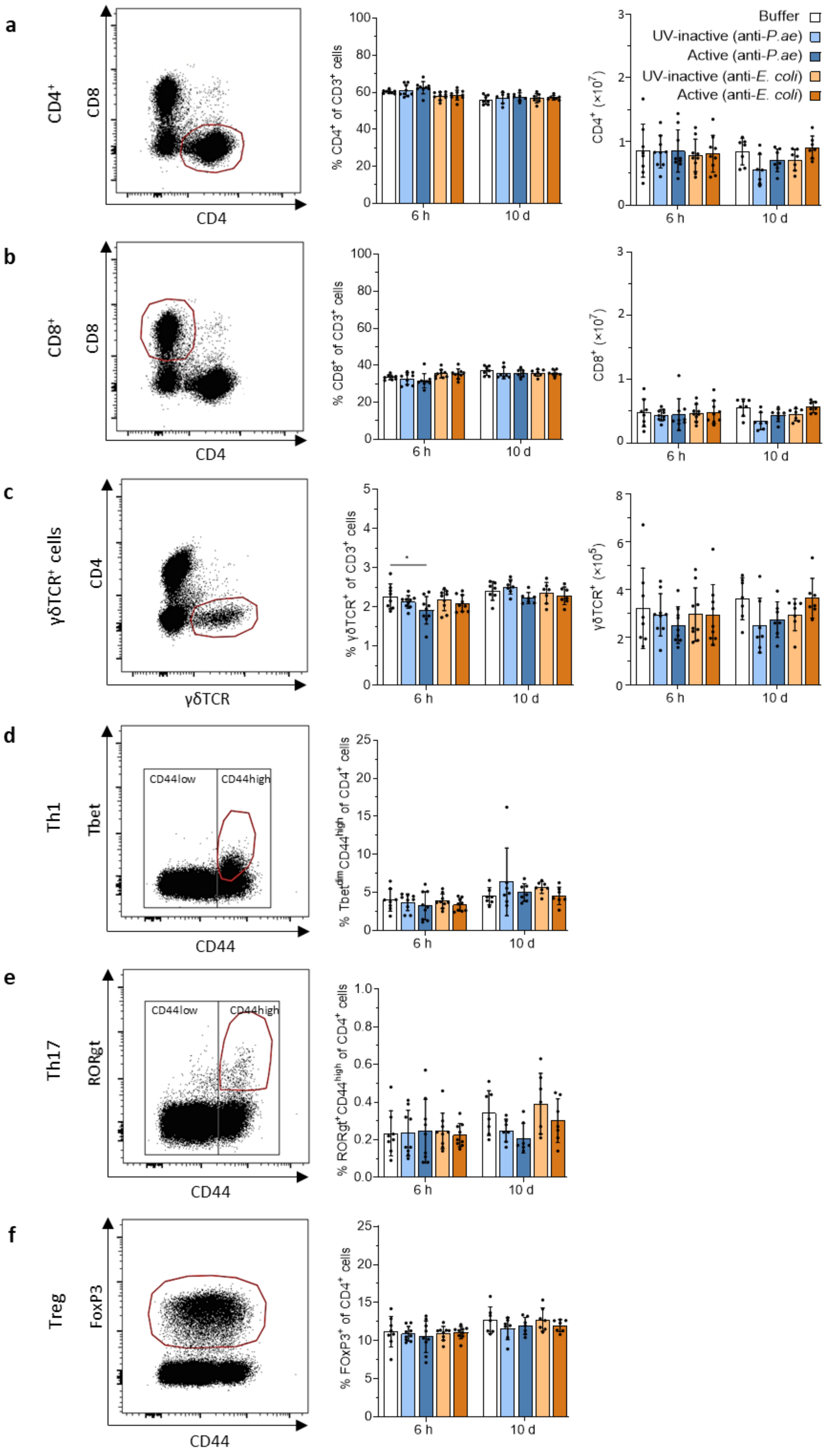

Supplementary Materials

Figure S5. T cell populations in the spleen show no marked changes after phage treatment. Analysis of (a) $CD4^+$, (b) $CD8^+$, (c) $\gamma\delta TCR^+$ T cells and the effector T cell subsets (d) Th1, (e) Th17 and (f) Treg in the spleen. Shown are representative dot plots (left) and bar graphs depicting the percentage of remaining cells (middle) or total cells (right). Results are shown as mean ± SD, as determined by 2-way ANOVA with Tukey´s multiple comparisons test: *$p < 0.05$. n = 7 – 9 mice per group. For full gating strategy see Figure S2.

Supplementary Materials

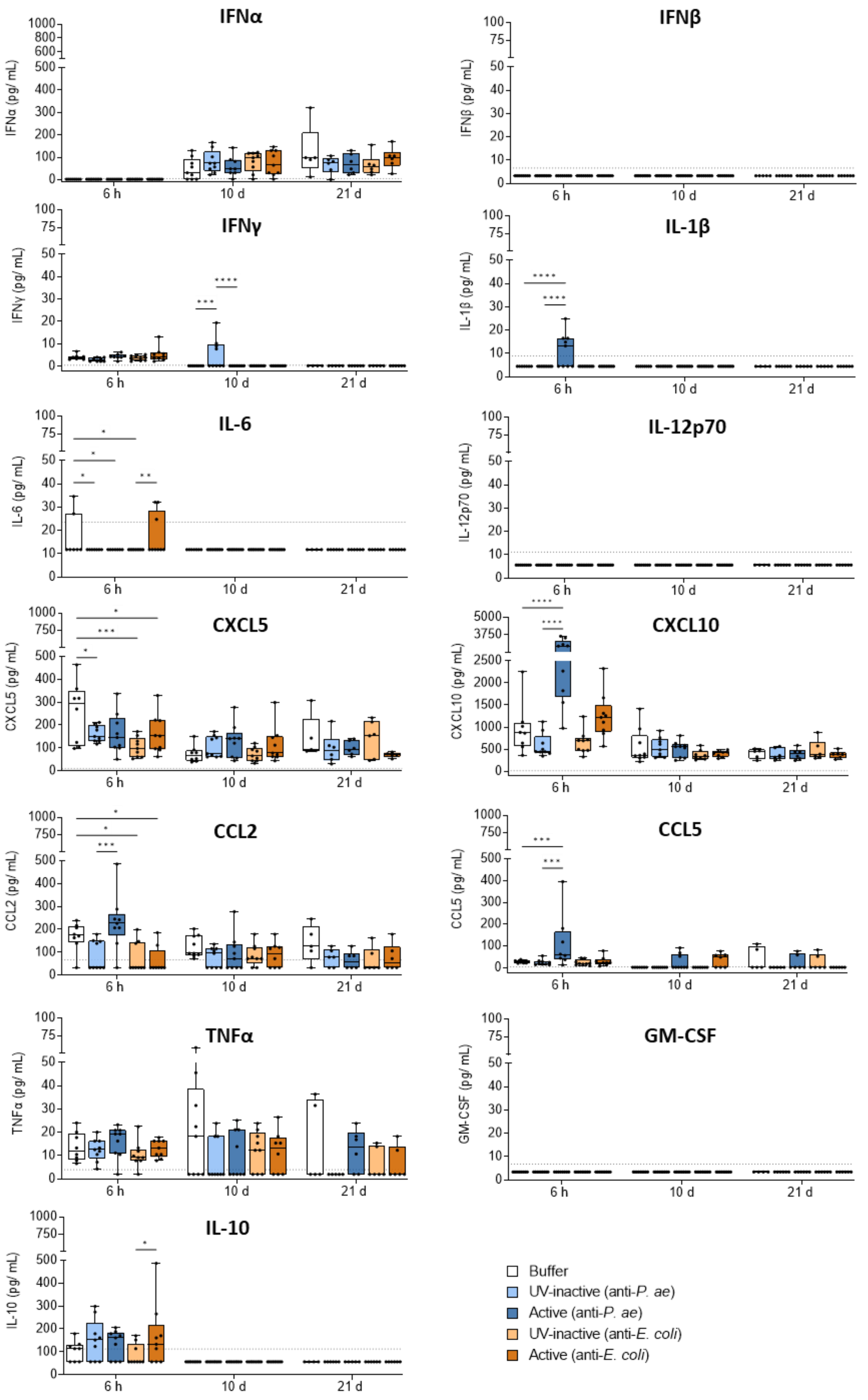

Supplementary Materials

Figure S6. Minimal cytokine release in response to phage treatment**.** Soluble cytokines and chemokines (pg/mL) in plasma at 6 h, 10 d and 21 d time points were measured via the Mouse Anti-Virus Response Panel (13-plex) from BioLegend. Results are shown as mean ± SD, as determined by 2-way ANOVA with Tukey´s multiple comparisons test: $*p < 0.05$; $**p < 0.01$; $***p < 0.001$. n = 5 – 9 mice per group. Dotted lines represent the detection limit, respectively.

Supplementary Materials

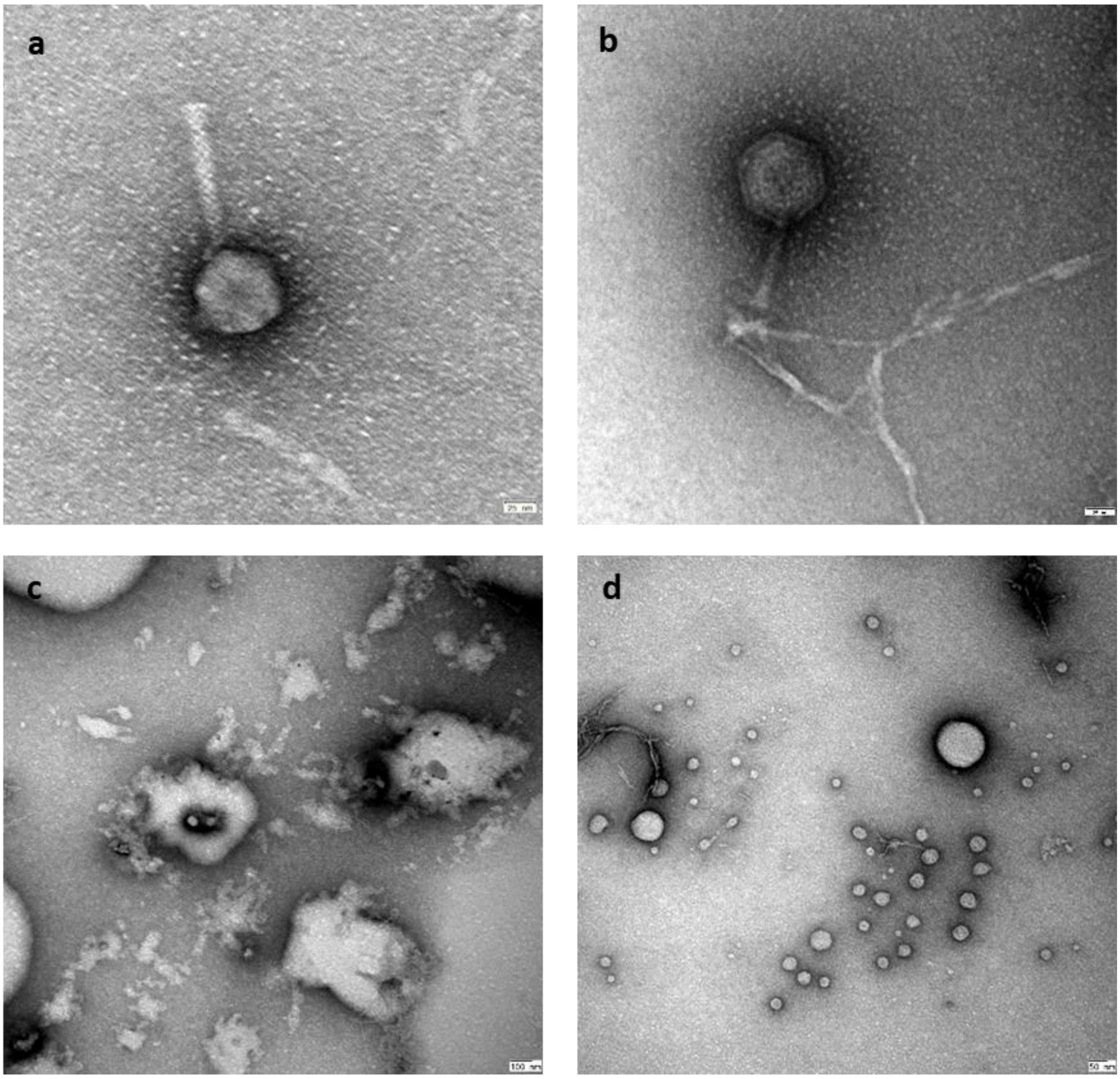

Figure S7. Negative staining transmission electron microscopic images of phage cocktails reveal that UV-inactivation may lead to destroyed phage structures. The two phage cocktails targeting *P. aeruginosa* (a, c) or *E. coli* (b, d) were analyzed via electron microscopy. In the active cocktails (a, b) the virus-like structure of the phages could be shown, whereas in the UV-inactivated samples (c, d) only artifacts could be found. Representative images with indicated bar scale (a – 25nm, b – 25nm, c – 100nm, d – 50nm).
